# Prediction of outcomes in patients with local recurrent nasopharyngeal carcinoma: development and validation of a four-factor prognostic model integrating baseline characteristics and [^18^F]FDG PET/CT parameters

**DOI:** 10.1007/s00330-022-09232-1

**Published:** 2022-11-24

**Authors:** Wen Dongxiang, Liu Liting, Liang Yujing, Luo Meijuan, Guo Shanshan, Xiong Longbin, Chen Yanzhou, Chen Meiling, Ning Kang, Mai Haiqiang, Tang Linquan, Chen Qiuyan

**Affiliations:** 1 Guangdong Key Laboratory of Nasopharyngeal Carcinoma Diagnosis and Therapy, Sun Yat-sen University Cancer Center, State Key Laboratory of Oncology in South China, Collaborative Innovation Center for Cancer Medicine, Guangzhou, Guangdong Province People’s Republic of China; 2 Department of Nasopharyngeal Carcinoma, Sun Yat-sen University Cancer Center, Dongfengdonglu 651, Guangzhou, Guangdong Province People’s Republic of China; 3 Department of Urology, Sun Yat-sen University Cancer Center, Guangzhou, Guangdong Province People’s Republic of China; 4 Department of Nuclear Medicine, Sun Yat-sen University Cancer Center, Guangzhou, Guangdong Province People’s Republic of China

**Keywords:** Positron emission tomography-computed tomography, Nasopharyngeal carcinoma, Recurrence, Nomogram, Prognosis

## Abstract

**Objectives:**

To investigate the prognostic value of [^18^F]FDG PET/CT parameters in local recurrent nasopharyngeal carcinoma (lrNPC) and establish a prognostic tool for lrNPC patients based on these [^18^F]FDG PET/CT parameters.

**Methods:**

A total of 358 lrNPC patients seen from 2010 to 2019 at Sun Yat-sen University Cancer Center with complete baseline characteristics and [^18^F]FDG PET/CT data were retrospectively analyzed. Maximal standardized uptake value (SUVmax), SUVmean, SUVpeak, metabolic tumor volume (MTV), total lesion glycolysis (TLG), and heterogeneity index (HI) for recurrent nasopharynx tumors were included. Cox regression analysis was performed to select candidate variables. Subsequently, a nomogram for predicting overall survival (OS) for lrNPC patients was developed and internally validated.

**Results:**

Multivariate Cox analysis results suggested that age ≥ 47 years (hazard ratio (HR), 1.62 (1.18-2.24); *p* = 0.003)，with smoking history (HR, 1.41 (1.01–1.98); *p* = 0.046), recurrent T stage {[rT3 vs rT1/2: HR, 1.81 (1.04–3.12); *p* = 0.037]; [rT4 vs rT1/2: HR, 2.46 (1.32–4.60); *p* = 0.005]}, and TLG {[37.1–184.3 vs ≤ 37.1: HR, 2.26 (1.49–3.42); *p* < 0.001]; [>184.3 vs ≤ 37.1: HR, 4.31 (2.50–7.43); *p* < 0.001]) were independent predictors of OS. A 4-factor nomogram was generated to stratify patients into 3 risk groups. This novel model showed good discrimination with a high C-index (0.752, 95%CI: 0.714–0.790). In addition, the calibration curves showed good agreement between the predicted probabilities and actual observations and decision curve analysis (DCA) suggested that the nomogram was useful for clinical decision-making.

**Conclusions:**

Our study confirmed that [^18^F]FDG PET/CT parameters were valuable in predicting OS and PFS for lrNPC patients. The 4-factor prognostic model combing baseline patient characteristics with [^18^F]FDG PET/CT parameters for lrNPC patients had good discrimination, agreement, and clinical application potential.

**Key Points:**

*•* *[*^*18*^*F]FDG* *PET/CT* *parameters were valuable in predicting OS and PFS for lrNPC patients.*

*• The novel 4-factor nomogram for lrNPC patients had good discrimination, agreement, and potential for clinical application.*

**Supplementary Information:**

The online version contains supplementary material available at 10.1007/s00330-022-09232-1.

## Introduction

Nasopharyngeal carcinoma (NPC) is a head and neck cancer with a distinct geographical distribution that is particularly prevalent in South China, Southeastern Asia, and North Africa [1]. Although intensity-modulated radiotherapy (IMRT) has attained excellent locoregional control rates, approximately 10% of NPC patients will experience local and/or regional lymph node recurrence after following primary treatment [2, 3]. Currently, local reirradiation or surgery achieves a good curative effect and significantly increases the overall survival (OS) of these patients [4–8]. However, for some lrNPC patients, the prognosis remains relatively poor, or serious side effects are experienced. Therefore, it is urgent in clinical practice to identify the different risk levels of patients for individualized treatment [2, 9].

Clinically, many guidelines recommend performing [^18^F]FDG PET/CT at the time of local recurrence because up to 20% of lrNPC patients have been reported to have concomitant distant metastasis [10–13]. In previous studies, some [^18^F]FDG PET/CT parameters, specifically, maximal standardized uptake value (SUVmax), metabolic tumor volume (MTV), and total lesion glycolysis (TLG) were correlated with clinical outcomes for various cancers [14–16]. Previous models for prognostic stratification of recurrent NPC patients were established based on the gross tumor volume (GTV), prior radiotherapy-induced grade, synchronous nodal recurrence, the EBV-DNA level and baseline characteristics including age, physical state, hypertension, and the recurrent T-category [17–20]. However, it is unclear whether these [^18^F]FDG PET/CT parameters can predict the prognosis of patients with lrNPC. Therefore, our current study aimed to elucidate the role of these parameters in lrNPC and establish a survival model that combined these parameters with other important clinical prognosticators for tailoring lrNPC patients and tailoring individual therapy.

In our study, we aimed to investigate the prognostic value of [^18^F]FDG PET/CT parameters for predicting the OS and PFS of lrNPC patients, and the correlation between these parameters and clinically important factors of the recurrent TNM stage and the Epstein-Barr virus DNA (EBV-DNA) level. In addition, we intended to develop and evaluate an OS nomogram integrating PET/CT parameters and baseline characteristics for predicting the prognosis of lrNPC.

## Materials and methods

### Patient inclusion criteria

A total of 358 lrNPC patients were treated at Sun Yat-sen University Cancer Center (SYSUCC) from Nov 2010 to May 2019 with a complete pre-therapeutic baseline and ^18^F-FDG PET/CT data were retrospectively reviewed. Inclusion criteria were (1) lrNPC with or without regional lymph nodal metastasis, (2) no evidence of distant metastasis, (3) aged 18 to 70 years, and (4) diagnosis confirmed by either pathology or radiological findings and clinical symptoms. In addition, [^18^F]FDG PET/CT was performed less than 2 weeks before lrNPC treatment and a minimum of 24-month follow-up.

### Data collection and ethics

Demographics and clinical information, such as age, sex, smoking history, drinking history, hypertension, NPC family history, ECOG performance score, prior treatments, recurrent TNM stage (the 8th Edition of the Union for International Cancer Control TNM staging system), and pre-treatment plasma EBV-DNA levels were collected. This study was approved by the Ethics Committee of Sun Yat-Sen University Cancer Center Health Authority (approved number: B2022-055-01) and was performed according to the ethical standards of the Declaration of Helsinki (as revised in 2013). Reporting of the study conforms to STROBE, along with references to STROBE and the broader EQUATOR guidelines. The study data underlying the findings of the current work were deposited at the Research Data Deposit platform (available at http://www.researchdata.org.cn/).

### PET/CT image acquisition and analysis

[^18^F]FDG PET/CT scans were performed using a dedicated PET/CT system (Discovery ST16; GE Medical Systems, Milwaukee, WI, USA). Imaging was performed using a combination PET/CT scanner according to PET/CT tumor imaging guidelines [21]. Detailed information on PET/CT protocol was described in the previous study [22]. The reconstruction was performed using the ordered subset expectation maximization iterative algorithm (OSEM). [^18^F]FDG PET/CT parameters, including SUVmax, SUVpeak, SUVmean, and MTV were obtained from [^18^F]FDG PET images of the recurrent nasopharynx tumor using PMOD software (PMOD Technologies Ltd.). As in previous studies, an SUV of 2.5 was used as a threshold for lesion contouring [23, 24], and contours around the target lesion inside the boundaries were automatically generated. MTV was recorded at an SUV > 2.5 within the contouring margin, while benign lesions were excluded. Lastly, TLG was calculated as SUVmean × MTV [25], and HI was calculated as SUVmax/SUVmean [26].

### Nomogram development, evaluation, and validation

Univariate Cox regression analyses were used to select candidate clinical predictors using a significant threshold of *p* < 0.1. Multivariable Cox analysis was performed to confirm the independent factors for the OS of lrNPC patients using the forward stepwise method. A nomogram was then developed based on this multivariate Cox proportional risk regression model. The discrimination of the model was evaluated using the Harrell Concordance Index (C-index). A Receiver operating characteristic (ROC) curve analysis was used to evaluate the sensitivity and specificity of this nomogram. Subsequently, the calibration plots of the nomogram were assessed by comparing the observed Kaplan-Meier estimates of survival probability to the nomogram-predicted survival probability. DCA curves were generated to assess the clinical application of the model. The predictive performance of the final model was internally validated using two-step bootstrap resampling procedures.

### Statistical analysis

The primary outcome was OS, defined as the time from lrNPC diagnosis to all-cause death or censoring at the date of the last follow-up. Progression-free survival (PFS) was defined as the duration from the diagnosis date of lrNPC to the date of disease progression according to the RECIST 1.1 guidelines or all-cause death. The case deletion method was used to handle missing values in all explanatory variables. The best cut-off values of continuous variables were calculated by X-tile (Version3.6.1). Continuous variables are expressed as medians (IQRs), and categorical variables are expressed as numbers (percentages). The unpaired continuous variable data were compared using the Mann-Whitney test. These differential parameter levels at different clinical stages analysis used a one-way ANOVA. A linear correlation analysis was used to analyse the correlation between EBV-DNA levels and these 6 parameters. Associations between OS and potential prognostic factors were assessed by using the log-rank test in univariate analysis. All statistical analyses were undertaken by using R version-4.0.5, SPSS version-25.0, and GraphPad Prism version 9. All *p* < 0.05 (two-tailed) was considered statistically significant.

## Results

### Patient characteristics

Of the 358 patients included in this study. 258 were males, 100 were females, and the median age was 47 years (IQR: 40–55 years). The median values of SUVmax, SUVpeak, SUVmean, MTV, TLG, and HI were 10.1 g/mL (IQR: 6.6–14.3 g/mL), 6.8 g/mL (IQR: 4.7–10.4 g/mL), 4.2 g/mL (IQR: 3.6–5.1 g/mL), 9.5 mL (IQR:3.8–24.6 mL), 42.9 g/mL × mL (IQR: 14.8–117.0 g/mL × mL), and 2.3 (IQR: 1.8–2.7), respectively. The median follow-up time was 37.2 (IQR: 26.0–50.7) months. The median OS and PFS were 56.3 (IQR: 47.0–65.6) and 36.5 (IQR: 31.5–41.5) months, respectively. The 1-, 3-, and 5-year OS (PFS) rates were 90.2% (81.8%), 67.9% (50.5%), and 48.1% (31.5%), respectively. Table 1 demonstrated other patients’ characteristics, including smoking/drinking/NPC family history, ECOG performance score, comorbidity, EBV DNA levels, recurrent T/N/overall stage, and treatments. In addition, the causes of death are provided in Supplementary Results.

**Table 1:** Patient characteristics

Patients	*n* = 358
Age (years), median (IQR)	47 (40–55)
Male, *n* (%)	258 (72.1%)
Smoking history, *n* (%)	108 (30.2%)
Drinking history, *n* (%)	50 (14.0%)
ECOG performance score, *n* (%)	
0	317 (88.5%)
1	41 (11.5%)
Hypertension, *n* (%)	45 (12.6%)
NPC family history, *n* (%)	32 (8.9%)
With detectable EBV-DNA, *n* (%)	176 (49.2%)
Recurrent T stage, *n* (%)	
rT1	51 (14.2%)
rT2	35 (9.8%)
rT3	176 (49.2%)
rT4	96 (26.8%)
Recurrent N stage, *n* (%)	
rN0	257 (71.8%)
rN1	84 (23.4%)
rN2	11 (3.1%)
rN3	6 (1.7%)
Recurrent overall stage, *n* (%)	
Stage I	35 (9.8%)
Stage II	50 (14.0%)
Stage III	172 (48.0%)
Stage IV	101 (28.2%)
Treatment of primary NPC, *n* (%)	
Radiotherapy	37 (10.3%)
CCRT	124 (34.6%)
IC+CCRT	118 (33.0%)
CCRT+AC	67 (18.7%)
IC+CCRT+AC	12 (3.4%)
Treatment of lrNPC, *n* (%)	
Surgery	78 (21.8%)
Radiotherapy	48 (13.4%)
Surgery/radiotherapy with chemotherapy/target therapy	189 (52.8%)
Palliative chemotherapy	43 (12.0%)
SUVmax (g/mL), median (IQR)	10.1 (6.6–14.3)
SUVpeak (g/mL), median (IQR)	6.8 (4.7–10.4)
SUVmean (g/mL), median (IQR)	4.2 (3.6–5.1)
MTV (mL), median (IQR)	9.5 (3.8–24.6)
TLG (g/mL × mL), median (IQR)	42.9 (14.8–117.0)
HI, median (IQR)	2.3 (1.8–2.7)

Abbreviations: *AC* adjuvant chemotherapy, *BMI* body mass index, *CCRT* concurrent chemoradiotherapy, *EBV* Epstein–Barr virus, *ECOG* The Eastern Cooperative Oncology Group, *HI* heterogeneity index, *IC* induction chemotherapy, *IQR* interquartile range, *lrNPC* local recurrent nasopharyngeal carcinoma, *MTV* metabolic tumor volume, *SUVmax* maximum standardized uptake value, *SUVmean* mean standardized uptake value, *SUVpeak* peak standardized uptake value, *TLG* total lesion glycolysis

### Correlation analysis

**Fig. 1:**
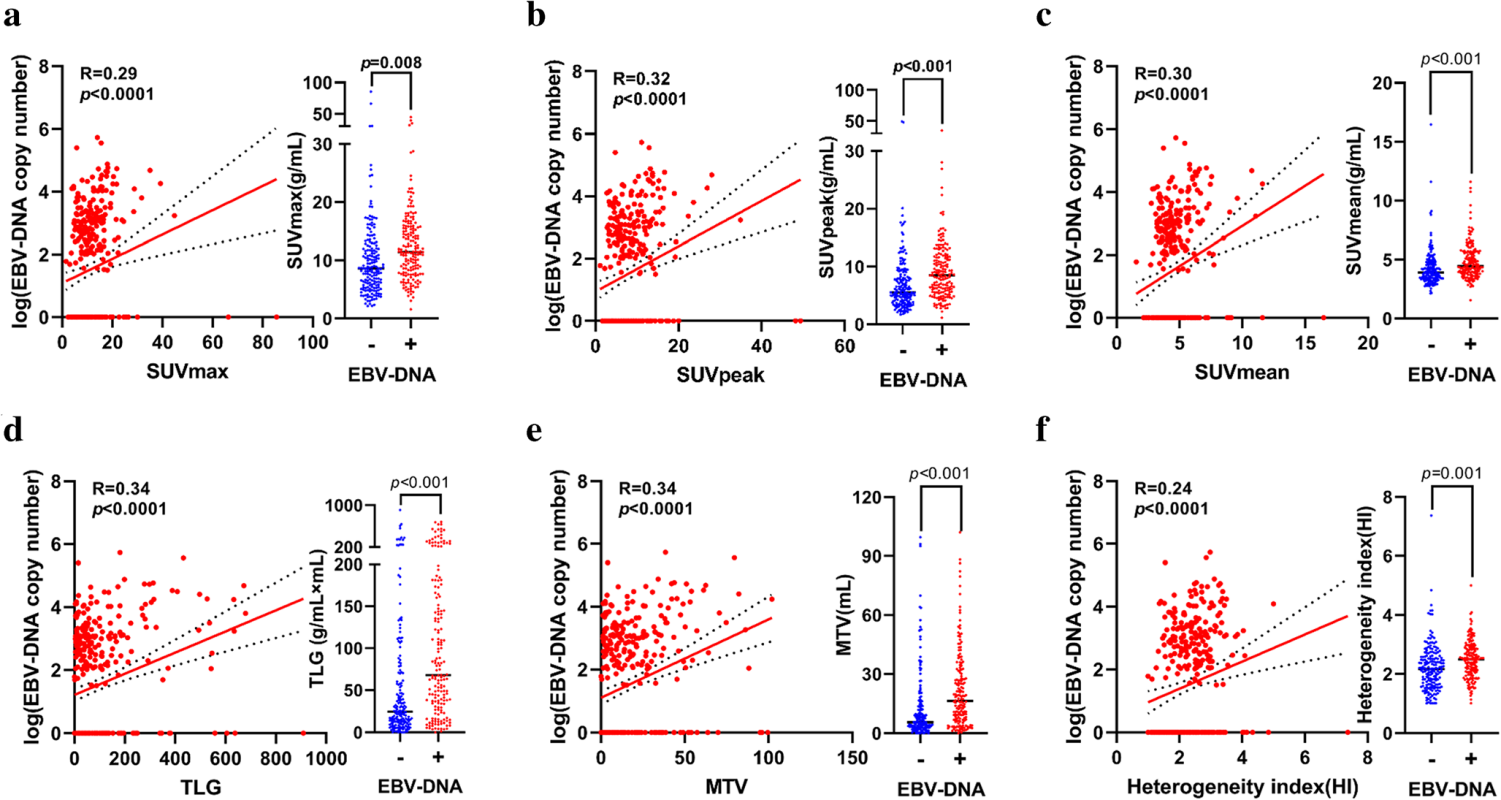
Correlation between [^18^F]FDG PET/CT parameters and EBV DNA. Pretherapeutic plasma EBV DNA level related to SUVmax (**a**), SUVpeak (**a**), SUVmean (**c**), TLG (**d**), MTV (**e**), and HI (**f**). SUVmax: maximal standardized uptake value; TLG: total lesion glycolysis; HI: heterogeneity index

Correlations between [^18^F]FDG PET/CT parameters and clinical characteristics such as recurrent T stage, N stage, overall stage, and EBV-DNA levels (log_10_) were calculated. As shown in Figure S1, the more advanced the recurrent T stage and overall stage were, the higher the levels of these 6 PET/CT parameters (SUVmax, SUVpeak, SUVmean, TLG, MTV, and HI) were (all *p* < 0.0001). However, in the advanced recurrent N stage, only TLG and MTV increased with the advanced stage, possibly due to the number of patients with recurrent N3 (Figure S1G-L). Linear correlation analysis indicated that these 6 parameters were positively related to EBV-DNA level (R = 0.24–0.34, all *p* < 0.0001, Fig. 1A–F). In addition, patients with detectable EBV-DNA levels (EBV-DNA copy number > 0) had higher levels of these 6 parameters than patients with undetectable EBV-DNA levels (Fig. 1A–F, all *p* < 0.05).

### Prognostic analysis

**Fig. 2:**
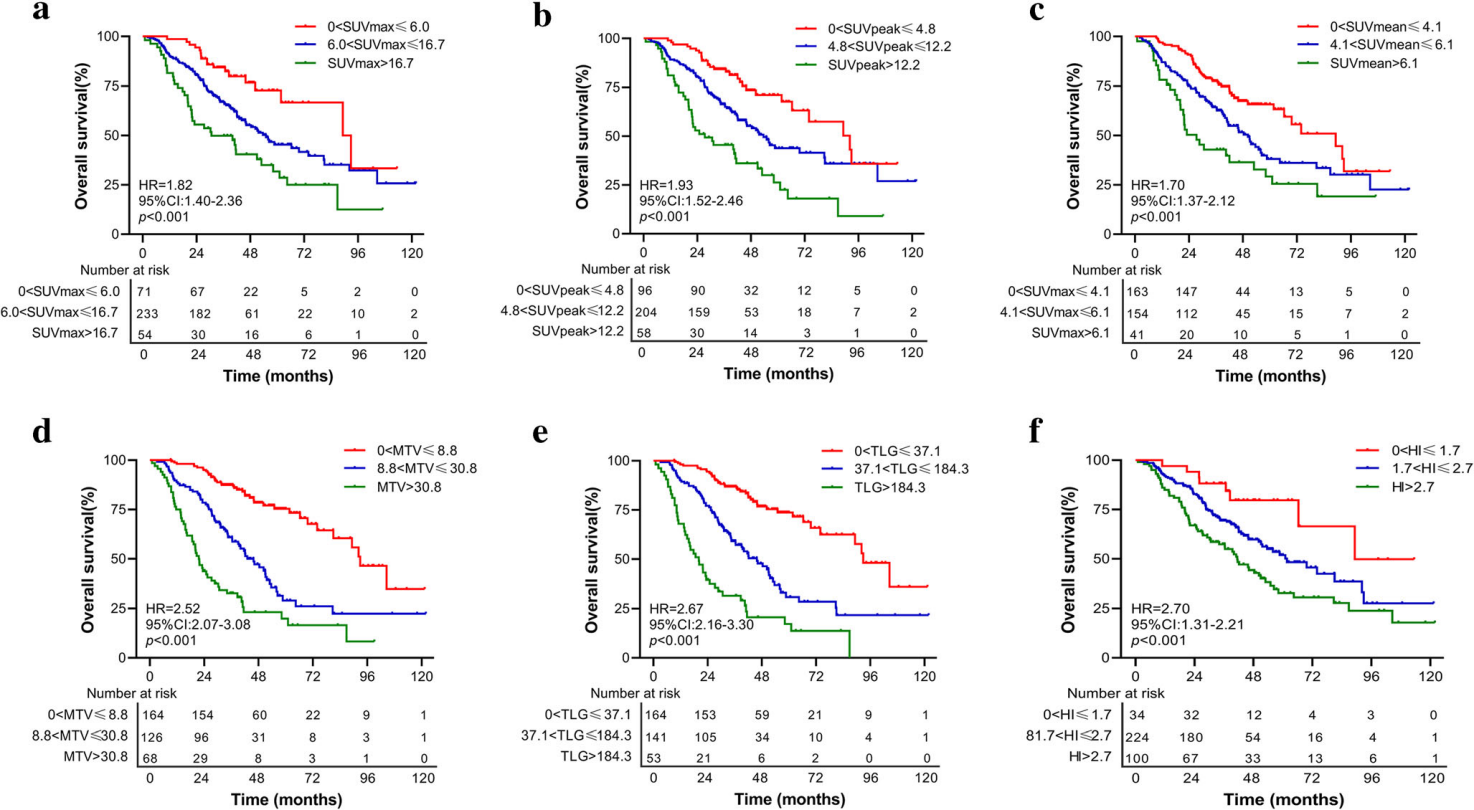
Kaplan–Meier survival curves comparing overall survival (OS) stratified by the cut-off SUVmax (**a**), SUVpeak (**b**), SUVmean (**c**), MTV (**d**), TLG (**e**), and HI (**f**). *p* values were calculated using the log-rank test. SUV: maximal standardized uptake value, MTV: metabolic tumor volume, TLG: total lesion glycolysis, HI: heterogeneity index

Univariate Cox regression analyses were performed to estimate the prognostic value of these PET/CT parameters in patients with lrNPC. As shown in Fig. 2 and Figure S2, these pretreatment PET/CT parameters included SUVmax, SUVpeak, SUVmean, MTV, TLG and HI were all associated with OS and PFS (all *p* < 0.05). The higher levels of these 6 parameters were, the worse the OS and PFS of lrNPC patients. In addition, some patients’ characteristics were also analyzed (Supplementary Results and Figure S3-4). Table 2 demonstrates bootstrap validation of the univariate Cox regression for PET/CT parameters and patient characteristics.

**Table 2:** Univariate Cox regression analyses and bootstrap validation in lrNPC patients

Variable	Reference	Bootstrap		
		HR	95% CI	Counts (*n*/500)
Age (years)	< 47	1.75	1.73–1.77	399
Smoking history	No	1.63	1.60–1.65	350
Hypertension	No	1.79	1.75–1.83	308
Recurrent T stage				
rT3	T1/2	2.88	2.80–2.95	442
rT4		6.21	6.05–6.36	500
Recurrent overall stage		2.29	2.26–2.31	500
Stage III	Stage I/II	2.86	2.78–2.93	436
Stage IV		5.88	5.73–6.03	499
EBV-DNA	Undetectable	1.52	1.49–1.54	292
Treatment				
Surgery	PCT	0.19	0.18–0.19	500
Radiotherapy		0.40	0.39–0.41	241
Surgery/RT+CT/TT		0.41	0.40–0.42	294
SUVmax (g/mL)				
6.0–16.7	≤ 6.0	2.15	2.09–2.20	339
> 16.7		3.69	3.57–3.81	466
SUVpeak (g/mL)				
4.8–12.2	≤ 4.8	1.99	1.95–2.02	364
> 12.2		3.94	3.84–4.04	492
SUVmean (g/mL)				
4.1–6.1	≤ 4.1	1.82	1.79–1.85	390
> 6.1		3.04	2.95–3.13	466
TLG (g/mL × mL)				
37.1–184.3	≤ 37.1	3.08	3.01–3.14	494
> 184.3		7.61	7.41–7.80	500
MTV (mL)				
8.8–30.8	≤ 8.8	3.18	3.12–3.25	492
> 30.8		6.89	6.70–7.07	500
HI				
1.7–2.7	≤ 1.7	2.35	2.27–2.43	141
> 2.7		3.75	3.61–3.88	399

Abbreviations: *95% CI* 95% confidence interval, *EBV* Epstein–Barr virus, *HI* heterogeneity index, *HR* hazard ratio, *IQR* interquartile range, *MTV* metabolic tumor volume, *NPC* nasopharyngeal carcinoma, *PCT* palliative chemotherapy, *RT+CT/TT* radiotherapy plus chemotherapy or target therapy, *SUVmax* maximum standardized uptake value, *SUVmean* mean standardized uptake value, *SUVpeak* peak standardized uptake value, *TLG* total lesion glycolysis

Subsequently, multivariate Cox regression was used to screen variables. As shown in Table 3, older (HR: 1.62, 95% CI: 1.18–2.24, *p* = 0.003), smoking history (HR: 1.41, 95% CI: 1.01–1.98, *p* = 0.046), advanced recurrent T stage (rT3 vs rT1/2: HR: 1.81, 95% CI: 1.04–3.12, *p* = 0.037; rT4 vs rT1/2: HR: 2.46, 95% CI: 1.32–4.60, *p* = 0.005) and a higher TLG value (37.1–184.3 vs ≤ 3.71: HR: 2.26, 95% CI: 1.49–3.42, *p* < 0.001; >184.3 vs ≤ 3.71: HR: 4.31, 95% CI: 2.50–7.43, *p* < 0.001) were identified as independent factors for worse OS of lrNPC patients.

**Table 3:** Multivariable Cox regression analyses and bootstrap validation in lrNPC patients

Variables	Multivariate Cox analysis			Model bootstrap validation			
	HR	95% CI	*p* value	HR	95% CI	Counts	*p* value
Age (years)	1.62	1.18–2.24	0.003	1.68	1.65–1.72	351	0.016
Smoking history	1.41	1.01–1.98	0.046	1.44	1.41–1.46	156	0.096
Recurrent T stage							
rT3 vs rT1/2	1.81	1.04–3.12	0.037	1.92	1.86–1.97	153	0.088
rT4 vs rT1/2	2.46	1.32–4.60	0.005	2.73	2.63–2.83	345	0.018
TLG (g/mL × mL)							
37.1–184.3 vs < 37.1	2.26	1.49–3.42	< 0.001	2.33	2.29–2.38	442	0.002
> 184.3 vs < 37.1	4.31	2.50–7.43	< 0.001	4.63	4.49–4.77	479	< 0.001

Abbreviations: *95% CI* 95% confidence interval, *HR* hazard ratio, *TLG* total lesion glycolysis

### Model establishment and validation

**Fig. 3:**
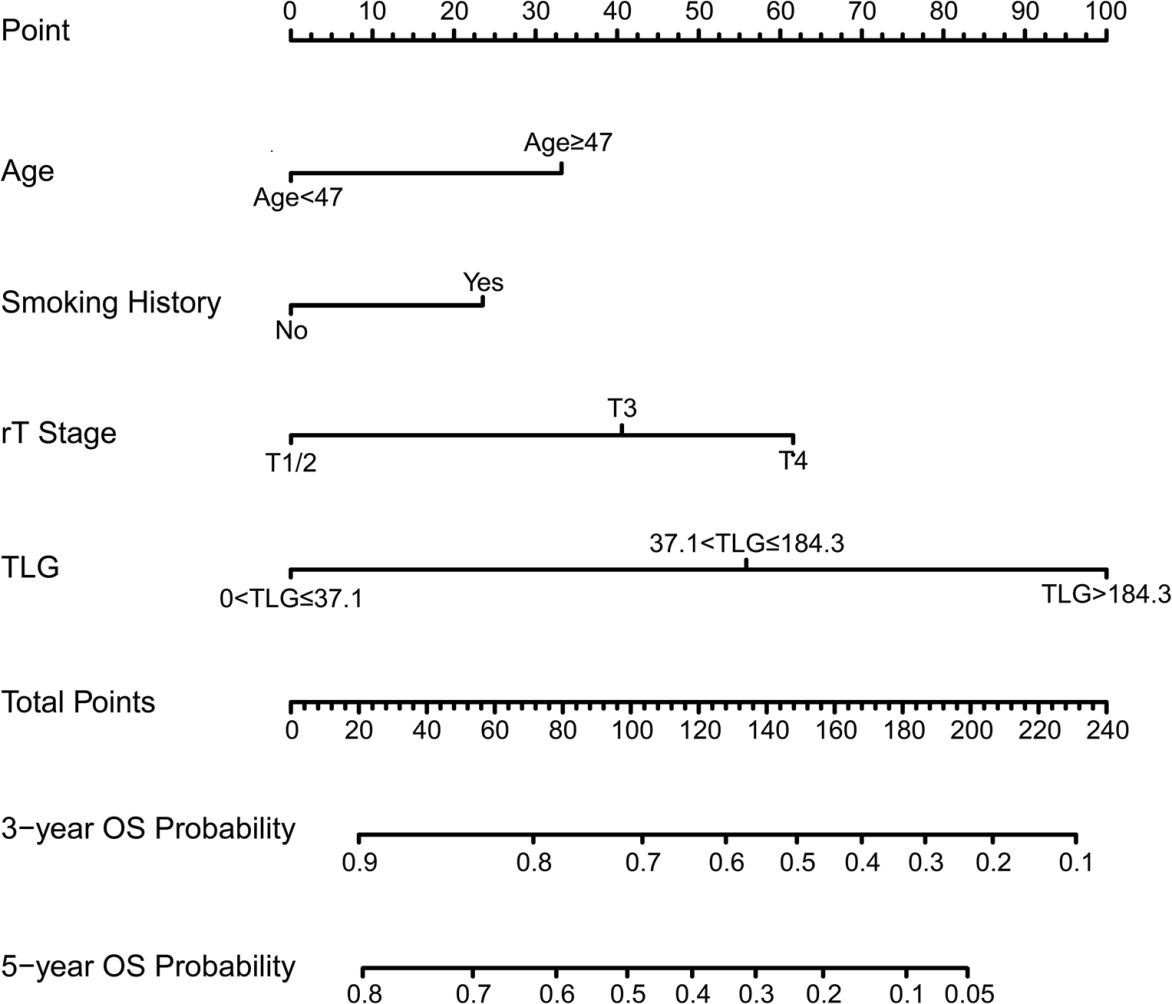
Prognostic nomogram for 3- and 5-year overall survival (OS) in patients with lrNPC. TLG: total lesion glycolysis

**Fig. 4:**
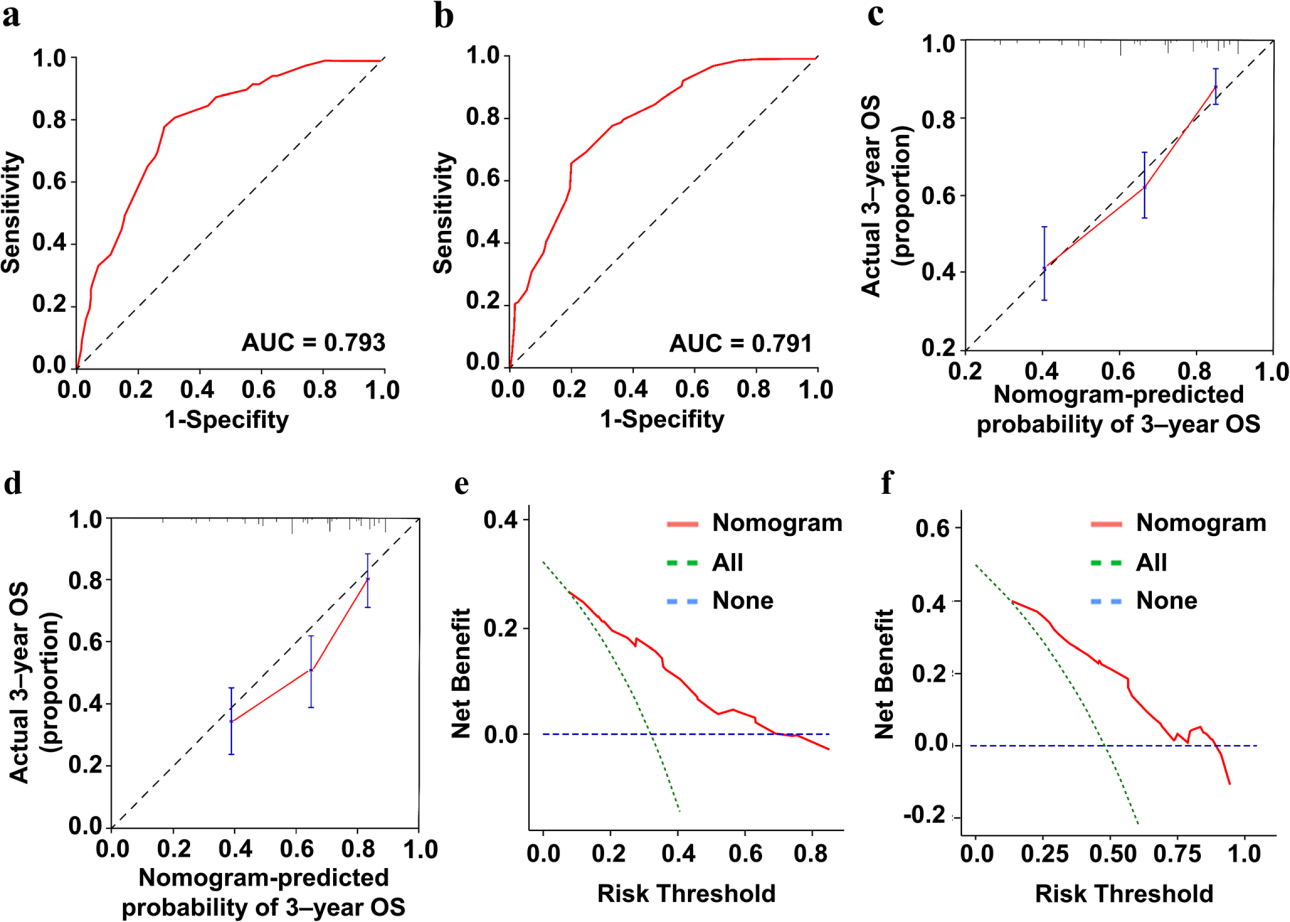
The calibration curve of the nomogram for predicting the 3-year (**c**) and 5-year (**d**) overall survival. Decision curves of the nomogram for the 3-year (**e**) and 5-year (**f**) overall survival

These four indicators of age, smoking history, recurrent T stage, and TLG were selected for our model. Using these four regression coefficients, an OS nomogram for individualized 3-year OS estimation was developed (Fig. 3). Every predictor variable value was assigned a corresponding score according to a point scale. By adding up the score of each variable and locating the total score to the survival rate scale, OS probabilities could be estimated at the time points of 3 and 5 years. The C-index was 0.752 (95% CI: 0.714–0.790). The prognostic accuracy of the nomogram for 3- and 5-year OS was also assessed using ROC curves with areas under the curve (AUCs) of 0.793 and 0.791, respectively (Fig. 4A, B). The calibration curves of the nomogram for OS are shown in Fig. 4C, D which showed good agreement between the estimated outcomes and the observed outcomes. As shown in the DCA curves (Fig. 4E, F), using the nomogram to predict OS offered a net benefit over the “treat-none” or “treat-all” strategy, suggesting that the nomogram was useful in clinical decision-making.

### Risk group stratification

**Fig. 5:**
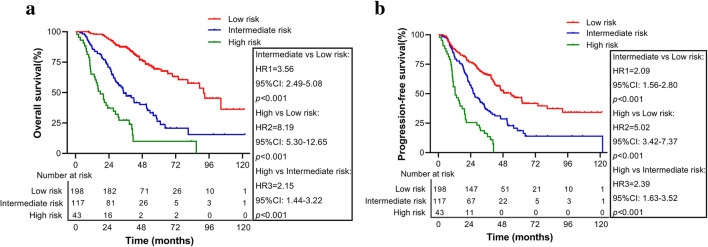
Kaplan-Meier survival curves of (**a**) overall survival (OS) and (**b**) progression-free survival (PFS) in different risk groups according to the total points scores

The risk stratification in our cohort was based on the best cut-off values of the total score derived from the nomogram model. All patients were categorized into three risk groups: low-risk (total score < 98), intermediate-risk (total score 98–162), and high-risk (total score >162). The median OS times of the low-, intermediate-, and high-risk groups were 92.2 (IQR: 75.9–108.4), 34.5 (IQR: 25.5–43.4), and 17.7 (IQR: 12.0–23.4) months, respectively. The median PFS times of the low, intermediate, and high-risk groups were 53.7 (IQR: 42.5–64.9), 26.5 (IQR: 21.4–31.7), and 12.5 (IQR: 7.5–17.5) months, respectively. In addition, the 3-year (5-year) OS rates were 87.5% (69.3%), 49.4% (26.3%), and 27.3% (9.9%) among these 3 risk groups, respectively. The 3-year (5-year) PFS rates were 66.3% (46.7%), 36.8% (18.7%), and 16.3% (0) in these 3 risk groups, respectively. Figure 5A shows the Kaplan–Meier survival curves for OS of different risk groups. The intermediate-risk (HR1: 3.56, 95%CI: 2.49–5.08, *p* < 0.001) and high-risk (HR2: 8.19, 95%CI: 5.30–12.65, *p* < 0.001) groups had a worse prognosis than the low-risk group, and the high-risk group also had a worse survival than the intermediate-risk group (HR3: 2.15, 95%CI: 1.44–3.22, *p* < 0.001). Similar results in PFS can be seen in Fig. 5B. Significant differences were also observed between the OS and PFS of patients with different risk levels, which further confirmed that our nomogram could appropriately stratify different risk lrNPC patients and may tailor individual therapy.

## Discussion

lrNPC presents a challenging treatment situation and the prognosis of patients is difficult to predict [5–7]. Therefore, developing a tool to stratify the prognosis of patients with lrNPC is of great clinical importance. In the clinic, it is routine for lrNPC patients to receive a [^18^F]FDG PET/CT since approximately 20% of patients have concomitant distant metastasis at the time of local recurrence [27, 28]. Previous studies have reported that PET/CT parameters such as SUVmax, MTV, and TLG may predict cancer prognosis [28–32]. Therefore, it is valuable to understand the correlation between PET/CT parameters and the prognosis of lrNPC. In this study, we found that higher PET/CT parameters, including SUVmax, SUVpeak, SUVmean, MTV, TLG, and HI, were significantly associated with advanced recurrent T stage, higher EBV-DNA levels, and worse OS and PFS of lrNPC patients. Based on these findings, a four-factor OS nomogram integrating age, smoking history, recurrent T stage, and TLG was developed and evaluated for predicting the prognosis of patients with lrNPC. This nomogram had good discrimination, calibration, and clinical application value. We also found that the nomogram could discriminate among high-risk, intermediate-risk, and low-risk patients with lrNPC. High-risk lrNPC patients may need more aggressive treatment, such as immunotherapy, because of the worst survival among different risk lrNPC patients.

[^18^F]FDG PET/CT, as the most widely used, functional imaging technique, can provide metabolic information for the entire tumor [29]. Furthermore, [^18^F]FDG FDG-PET has been shown to be superior to MRI in detecting residual/recurrent NPC with higher sensitivity, specificity, and accuracy [30]. The [^18^F]FDG standardized uptake value (SUV) has been reported to be associated with both the density of tumor cells and the glucose metabolic rate [31]. Lee SW et al found that NPC, which had a higher [^18^F]FDG uptake, had a significantly lower 3-year disease-free survival [32]. In addition, MTV and TLG derived from [^18^F]FDG PET/CT are strong predictors of OS in patients treated with radiotherapy, chemotherapy, and immunotherapy in several types of cancers [33–37]. Chan et al found that SUVmax can predict the surgical outcome of nasopharyngectomy and cervical lymphadenectomy for recurrent NPC [38]. Consistent with prior research, parameters including SUVmax, SUVpeak, SUVmean, MTV, TLG, and HI were found to be associated with the prognosis of lrNPC in our study (Fig. 1). Interestingly, these 6 parameters were also correlated with the recurrent T stage, as well as the serum EBV-DNA level, which was previously reported to be closely associated with the prognosis of NPC patients. TLG is a composite parameter representing tumor volume and metabolic status [39, 40]. In the multivariate Cox regression analysis of the current study, TLG was the only independent prognostic factor of [^18^F]FDG PCT/CT parameters for lrNPC patients.

In the era of intensity-modulated radiotherapy, approximately 10% of NPC patients still develop local or regional recurrence [3, 41]. Currently, the American Joint Committee Cancer (AJCC) recurrent TNM staging system (rTNM) is widely used to predict clinical outcomes for these patients. However, the usefulness of rTNM staging is limited, since these outcomes vary among patients within the same stage [6, 38, 42, 43]. Therefore, precise risk stratification to guide individualized treatment for these patients is an urgent clinical problem to be solved. Li et al constructed comprehensive prognostic models using age, GTV, prior RT-induced grade ≥ 3 toxicities, rT stage, and repeat IMRT equivalent dose in 2-Gy fractions (EQD2) to personalize recommendations for salvage intensity-modulated radiotherapy (IMRT) in patients with local recurrent NPC [19]. Similarly, Tian et al developed a prognostic-score model for classified local recurrent NPC patients suitable for undergoing reirradiation with IMRT. Model variables included Karnofsky performance status (KPS), age, late complications, recurrent T stage, synchronous nodal recurrence, and GTV [17]. Sun et al established a prognostic model integrating demographic and EBV-DNA levels [20]. However, this simple model did not have very good discrimination, with a C-index was 0.687, compared to ours of 0.752. Additionally, EBV-DNA measurement is difficult for many basic-level hospitals to achieve. In the current study, PET/CT parameters were strongly correlated with the prognosis of lrNPC (Fig. 2). However, no prognostic model based on these PET/CT parameters has been reported in the literature. Therefore, a prognostic model based on these parameters is valuable. Finally, we develop a 4-factor OS nomogram for lrNPC patients combining TLG and 3 patient characteristics including age, smoking history, and recurrent T stage. In our nomogram, smoking was one of the poor prognosis factors of lrNPC patients, which was consistent with previous reports [44, 45]. However, the poor results of the internal bootstrap validation may be due to the influence of sex on the sampling process.

Our study has several limitations. First, it was retrospective in design and therefore was prone to selection bias. Second, although our model exhibited good discrimination and agreement, the model lacked external validation. In addition, our model requires further validation in prospective studies and multicenter clinical trials.

The [^18^F]FDG PET/CT parameters were valuable in predicting OS and PFS in lrNPC patients, and our four-factor nomogram integrating clinical characteristics and [^18^F]FDG PET/CT parameters to predict OS for lrNPC patients had good discrimination, agreement, and potential for clinical application.

## Supplementary Information

ESM 1 (DOCX 1614 kb)

## Abbreviations

[^18^F]FDG: ^18^F-Fluoro-2-deoxy-2-D-glucose
AUC: Area under the curve
CI: Confidence intervals
C-index: Harrell concordance index
DCA: Decision curve analysis
EBV: Epstein-Barr virus
ECOG: The Eastern Cooperative Oncology Group
EQD2: Equivalent dose in 2-Gy fractions
GTV: Gross tumor volume
HI: Heterogeneity index
HR: Hazard ratio
IMRT: Intensity-modulated radiotherapy
KPS: Karnofsky performance status
LrNPC: Local recurrent nasopharyngeal carcinoma
MTV: Metabolic tumor volume
OS: Overall survival
PET/CT: positron emission tomography/computed tomography
PFS: Progression-free survival
RECIST: The response evaluation criteria in solid tumors
ROC: Receiver operating characteristic
SUVmax: Maximal standardized uptake value
SUVmean: Mean standardized uptake value
SUVpeak: Peak standardized uptake value
TLG: Total lesion glycolysis

## Declarations

### Guarantor

The scientific guarantor of this publication is Dr. Chen Qiuyan.

### Statistics and biometry

One of the authors has significant statistical expertise (Dr. Ning Kang).

### Methodology

• retrospective

• diagnostic or prognostic study

• performed at one institution
