# Supplementary material for: Prediction of outcomes in patients with local recurrent nasopharyngeal carcinoma: development and validation of a four-factor prognostic model integrating baseline characteristics and [18F]FDG PET/CT parameters
Source: Eur Radiol. 2022 Nov 24;33(4):2840–9. doi: 10.1007/s00330-022-09232-1 (PMC10017585; doi:10.1007/s00330-022-09232-1)
Supplement: Supplementary file 1 — (DOCX 1614 kb) [file 330_2022_9232_MOESM1_ESM.docx]

**Figure S1.** Correlation between ^18^F-FDG PET/CT parameters and clinical characteristics. rT stage related to SUVmax(A), SUVpeak(B), SUVmean(C), TLG(D), MTV(E), and HI(F); rN stage related to SUVmax(G), SUVpeak(H), SUVmean(I), TLG(J), MTV(K), and HI(L); recurrent overall stage related to SUVmax(M), SUVpeak(N), SUVmean(O), TLG(P), MTV(Q), and HI(R). SUVmax: maximal standardized uptake value; TLG: total lesion glycolysis; HI: heterogeneity index.


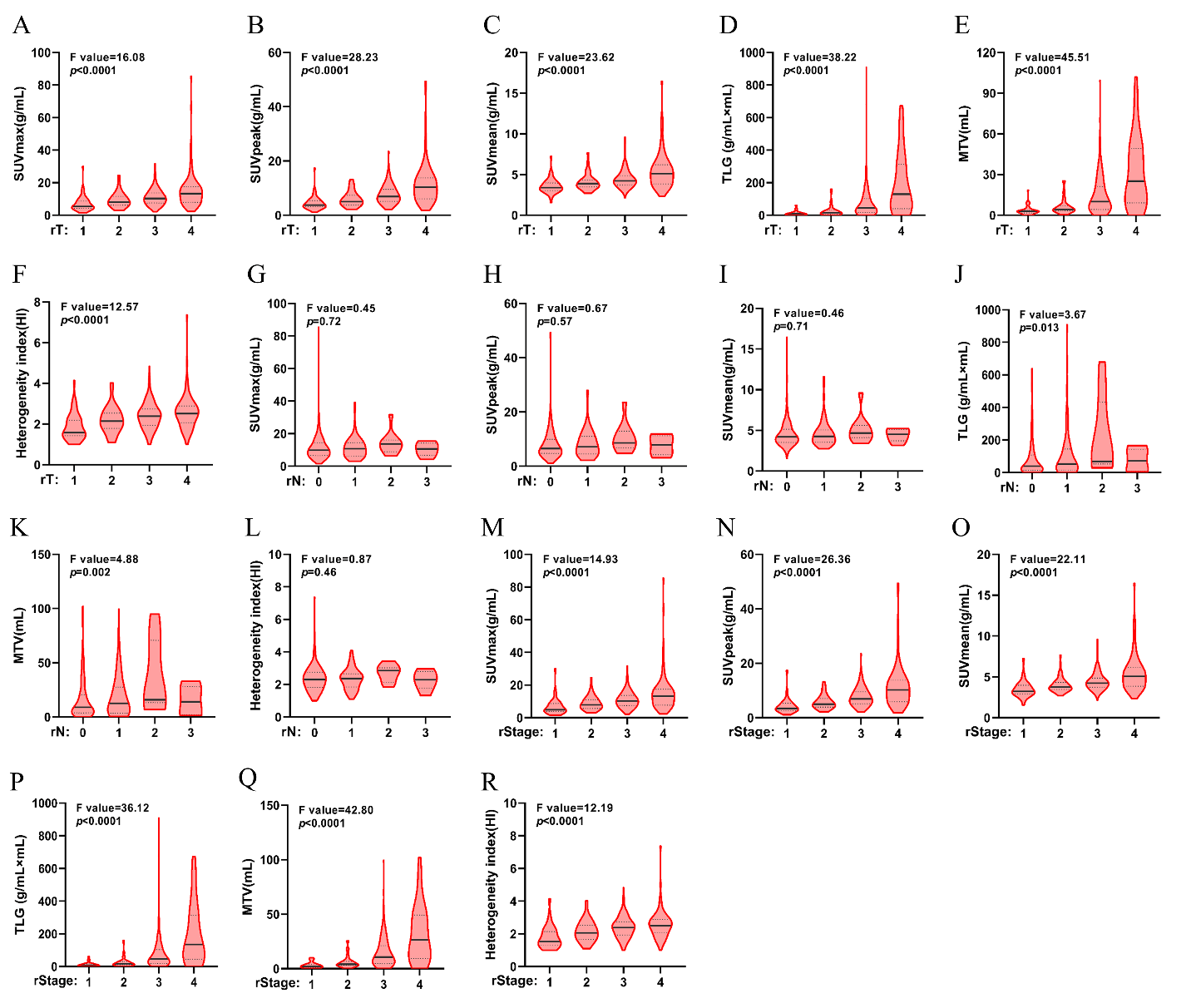


**Figure S2.** Kaplan–Meier survival curves comparing progression-free survival (PFS) stratified by the cut-off SUVmax (A), SUVpeak (B), SUVmean (C), MTV (D), TLG (E), and HI (F). *P*-values were calculated using the log-rank test. SUV: maximal standardized uptake value, MTV: metabolic tumor volume, TLG: total lesion glycolysis, HI: heterogeneity index.


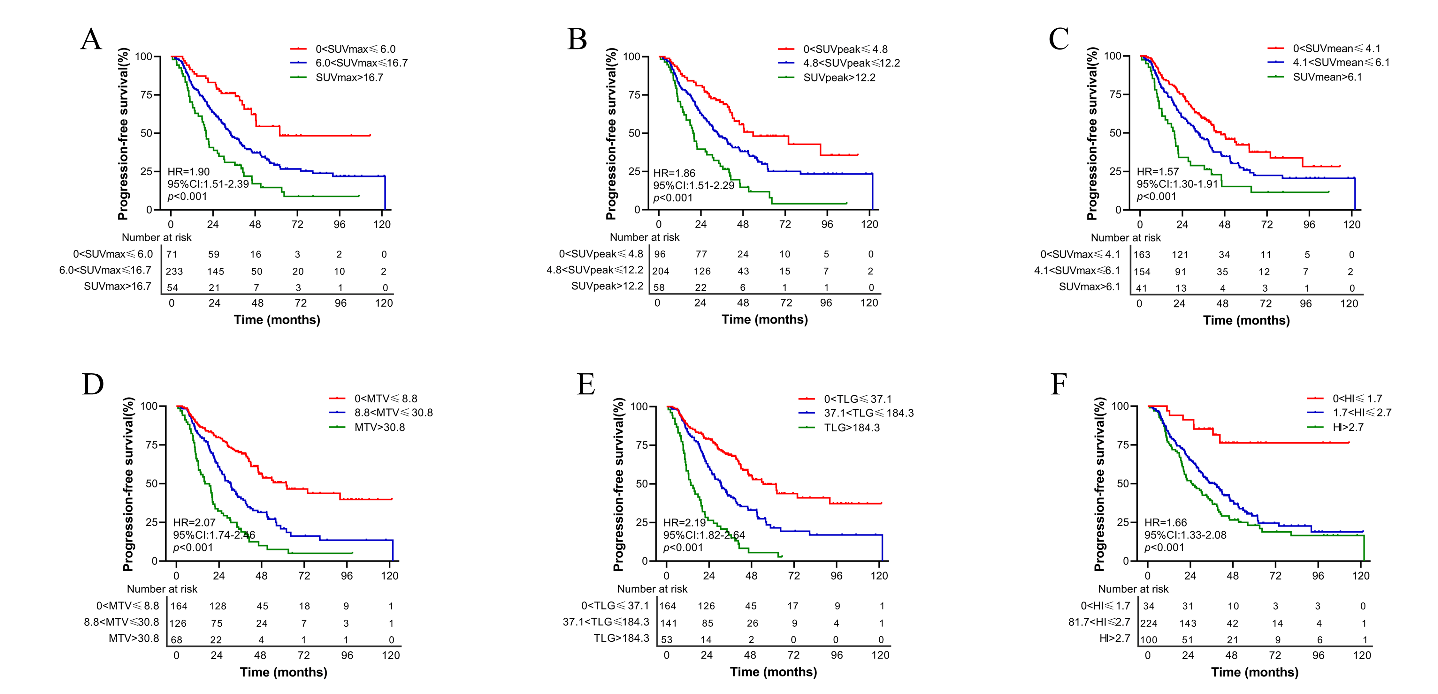


**Figure S3.** Kaplan–Meier survival curves comparing overall survival (OS) stratified by age (A), gender (B), ECOG status (C), smoking history (D), drinking history (E), hypertension (F), NPC family history (G), EBV-DNA level (H), rT stage (I), rN stage (J), recurrent overall stage (K), and treatment methods (L). *P* values were calculated using the log-rank test. EBV-DNA: Epstein-Barr virus DNA. ECOG: Eastern Cooperative Oncology Group; NPC: nasopharyngeal carcinoma; PCT: palliative chemotherapy. RT: radiotherapy; RT+CT/TT: radiotherapy plus chemotherapy/target therapy.


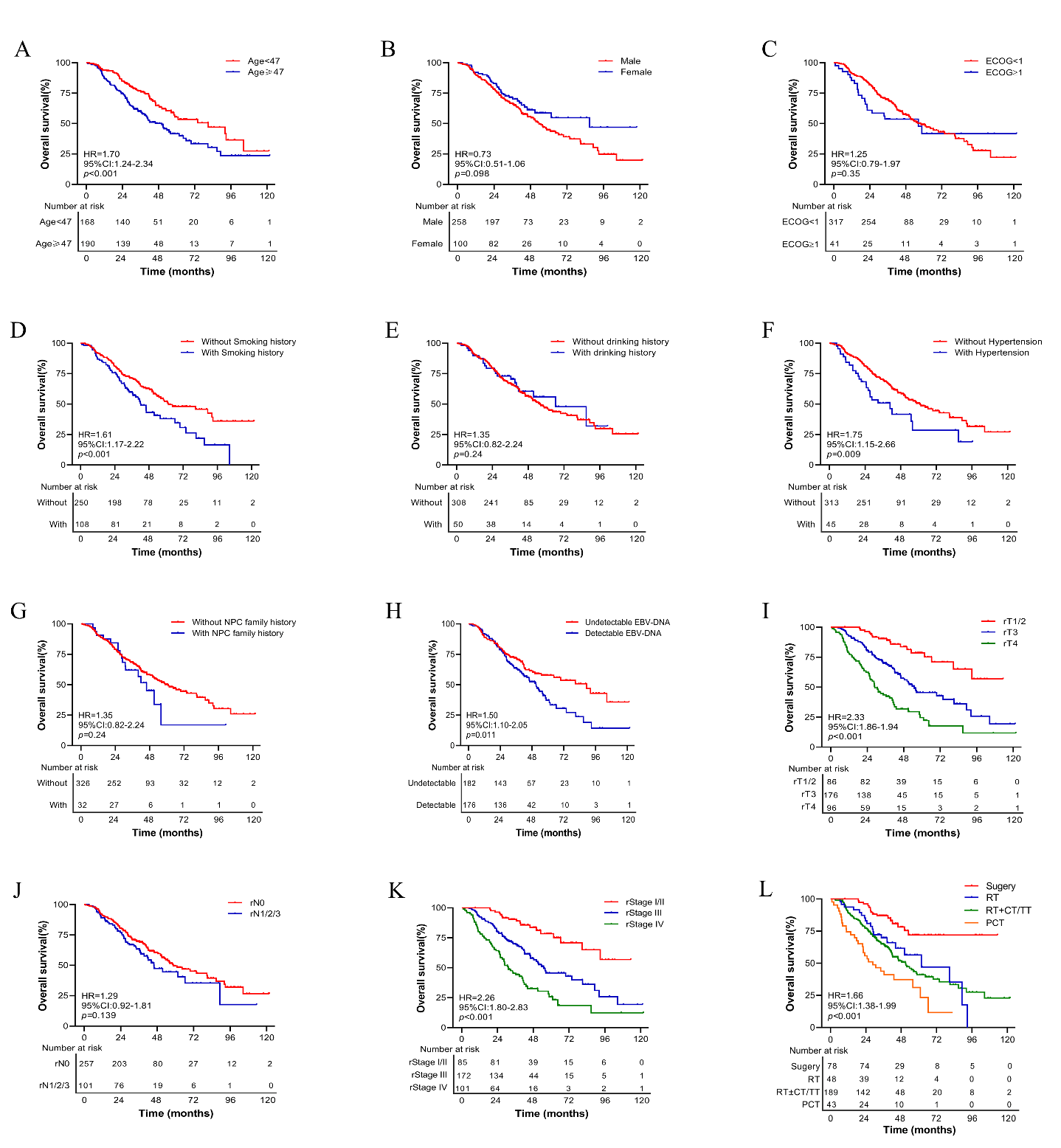


**Figure S4.** Kaplan–Meier survival curves comparing progression-free survival (PFS) stratified by age (A), gender (B), ECOG status (C), smoking history (D), drinking history (E), hypertension (F), NPC family history (G), EBV-DNA level (H), rT stage (I), rN stage (J), recurrent overall stage (K), and treatment methods (L). P values were calculated using the log-rank test. EBV-DNA: Epstein-Barr virus DNA. ECOG: Eastern Cooperative Oncology Group; NPC: nasopharyngeal carcinoma; PCT: palliative chemotherapy. RT: radiotherapy; RT+CT/TT: radiotherapy plus chemotherapy/target therapy.


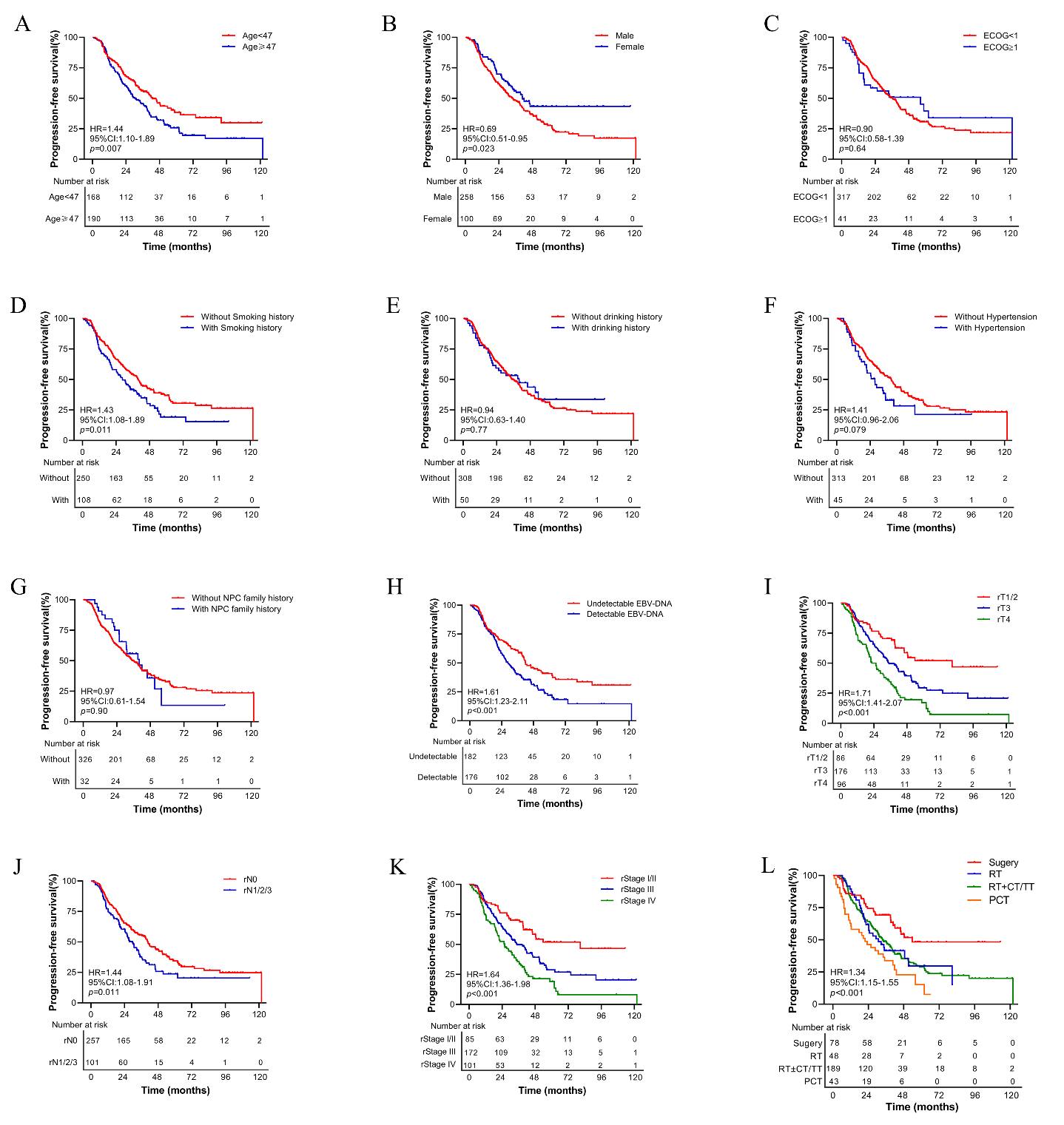


**Supplementary results**

**Causes of death**

A total of 221 (61.7%) patients progressed and/or died of any causes. Of the 162 (45.3%) patients who died during the follow-up: 110 (30.8%) and 28 (7.8%) died of local disease and distant metastasis, respectively. Additionally, 18 (5.0%) died of radiation-related complications (9 of nasopharyngeal necrosis or excessive nasal bleeding, 5 of radiation encephalopathy, and 4 of other radiation-related injuries), 1 (0.3%) died of respiratory disease, 1 (0.3%) died in an accident, and the remaining 4 (1.1%) patients had unknown causes.

**Prognostic analysis**

As shown in Figure S3 and Figure S4, age, smoking history, EBV-DNA level, recurrent T stage, overall stage and therapeutic methods were significantly related to both OS and PFS (all p<0.05). Patients with hypertension had worse PFS (p=0.009), while males and patients with lymph node metastasis had worse OS (p=0.023 and 0.011, respectively).. ECOG status, drinking history, and NPC family history failed to correlate with the OS and PFS of lrNPC patients (Figure S3 and Figure S4).
